# Numerical Modeling of a Sustainable Solid-State Recycling of Aluminum Scraps by Means of Friction Stir Extrusion Process

**DOI:** 10.3390/ma16124375

**Published:** 2023-06-14

**Authors:** Sara Bocchi, Gianluca D’Urso, Claudio Giardini

**Affiliations:** Department of Management, Information and Production Engineering, University of Bergamo, 24044 Dalmine, Italy; gianluca.d-urso@unibg.it (G.D.); claudio.giardini@unibg.it (C.G.)

**Keywords:** Friction Stir Extrusion, Finite Element Method, Piwnik and Plata

## Abstract

One of the most important purposes of the modern industry is a sustainable production, considering the minimization of the energy and of the raw materials used, together with the reduction of polluting emissions. In this context, Friction Stir Extrusion stands out, since it allows to obtain extrusions starting from metal scraps deriving from traditional mechanical machining processes (e.g., chips deriving from cutting operations), heated only by the friction generated between the scraps and the tool, so avoiding the material melting phase. Given the complexity of this new kind of process, the objective of this research is the study of the bonding conditions considering both the heat and the stresses generated during the process under different working parameters, namely tool rotational and descent speeds. As a result, the combined approach involving the Finite Element Analysis and the Piwnik and Plata criterion proves to be a valid tool for forecasting if bonding phenomenon occurs and how it is influenced by the process parameters. The results have also demonstrated that it is possible to achieve completely massive pieces between 500 rpm and 1200 rpm, but at different tool descent speeds. Specifically, up to 1.2 mm/s for 500 rpm and just over 2 mm/s for 1200 rpm.

## 1. Introduction

Over the past decades, the primary focus has been on resource use and efficiency to address environmental issues and promote environmentally and economically competitive energy resources. From here to now, the main goals are better resource efficiency and increased investment in technology development and in the green sector of resource recycling.

Regarding the aluminum, its traditional recycle process involves many and different passages, such as the re-melting of the scraps, the formation of new ingots, their necessary re-working to obtain new billets, and finally the real extrusion process. Moreover, the traditional fusion recovery of the metal scraps is not always possible due to: the high surface/volume ratio, the contaminating oil residues, and the covering alumina layer [1]. Indeed, the oxides trapped in aluminum generate internal stresses and significantly reduce its tensile strength, yield strength, and fatigue strength. Furthermore, the need to re-melt the metallic material in steel melting pots leads to the passage of a significant percentage of iron into solution within the molten aluminum. Iron is characterized by a low solubility within the solid aluminum matrix, approximately 0.04%, and this causes the precipitation of iron in intermetallic form within the new obtained alloy. These intermetallic precipitates are cathode to aluminum and thus lead to the formation of galvanic micro-cells with marked corrosion at the grain boundaries and the consequent nucleation, growth, and coalescence of the porosities [2]. For this reason, all the impurities should be eliminated before re-working an aluminum alloy; but, in general, complete elimination of these substances is difficult. Consequently, the alloys deriving from aluminum waste have a lower quality and a difficult to control composition [3]. Furthermore, conventional melt recycling technologies pose environmental, energy, economic, and technological problems [1]: to recover a metal, high temperatures must be reached through a combustion in an oven. As a result, there is evidence to date that conventional technologies do not meet the requirements of modern industry.

For this reason, in 1993, the Welding Institute developed a new process for recycling metal waste, known as Friction Stir Extrusion (FSE), especially used for the low melting metals, i.e., aluminum and magnesium [4,5,6], but also nowadays used for other materials, such as graphite, to built aluminum-graphite joining [7], copper [8], and copper-carbon composites [9]. With this process, it is possible to extrude new pieces just starting directly from the scraps, limiting the necessary phases of the recycle process. It is an energy-efficient technology used to synthesise and solid-state recycle different materials [10] mainly used for rather small pieces characterized by limited volume and mass. The novelty is to generate heat by friction between the scraps and the tool without requiring any external heating source. This consents great energy savings, considering that less than 15% of a conventional aluminum melting process energy is needed. The temperature reached by friction allows the softening of the material and, subsequently, the generation of a high plastic deformation useful for mixing, consolidating, and binding the scraps, obtaining extrusions characterized by good mechanical properties and eliminating the need for additional processing [11]. The FSE is an advanced solid-state technique for metal chips processing, since the reached temperature are around 400 °C, and it is a direct-recycling process, since there is no longer the need to recast the chip to recreate a new billet to be extruded, thus avoiding the problems typically due to the fusion processes: dendritic structure, cracks, hot oxidation, and shrinkage phenomena during solidification. Another important characteristic of the FSE process is the possibility to greatly refine the microstructure of the extruded pieces with respect to the traditional extrusion process [1,12,13].

During the FSE process, a rotating tool is immersed in a hollow matrix for compacting, stirring and, finally, extruding the recycled chips into a complete dense rod. The result is a cylindrical bar whose dimensions are subjected to both the diameter of the hole and the number of chips used, and which characteristics depend on the process parameters considered, in particular the tool rotational speed, responsible for the generation of heat, the descent speed of the tool, strictly correlated to the force exerted on the tool, and the size of the aluminum scraps [14,15,16]. As regards the force exerted by the descending tool, Tahmasbi and Mahmoodi demonstrated that the greater this force was, the more homogeneous was the cross section of the extrusion, with a resulting increase in the hardness of the piece [15]. Hosseini et al. demonstrated a correlation between the tool rotational speed and the quality of the obtained piece. A close correlation was highlighted between a low tool rotational speed and the presence of cold cracks on the external surfaces of the obtained pieces. This could be explained by the generation of an insufficient amount of heat to reach the softening temperature of the aluminum. Conversely, a high rotational speed can cause the formation of micro and macro porosity inside the products [17].

The development of simulative models is highly valuable for predicting the relationship between process parameters and the physics of Friction Stir Extrusion, which is a relatively new technology. An axisymmetric 2D model was developed to study the heat transfer model and to predict the temperature trend during the FSE, assuming that all the mechanical work was dissipated inside the billet, to heat it further. To do this, the container, the tool, the base on which the instrumentation was fixed, and the scraps as a single billet were all modelled. In this case, only a radial linear distribution of the heat flux was simulated [18]. Behnagh et al. modelled, with ABAQUS 2D Eulerian–Lagrangian finite element, the thermal model and the deformations undergone by magnesium scraps [16]. As a result of the mechanical, thermal, and microstructural analyses, it was noted by the authors that the tool rotational speed influenced the heat exchange much more than the tool descent speed. Baffari et al. studied the behavior of AZ31 magnesium products extruded with FSE with the final aim to predict the quality of the pieces obtained [19]. The model was based on three components: the tool, the container, and the piece, simulated as a single cylinder. Further, in this case, a relationship between component temperatures and process parameters was highlighted. In particular, the temperature is negatively influenced by the extrusion force and the working time. The higher the resistance of the material and the shorter the time, the lower the temperature reached by the material was. On the contrary, the tool rotational speed showed a positive influence: the temperature passed about from 250 °C with 500 rpm, to almost 500 °C with 900 rpm.

To the authors’ best knowledge, up to now, the analysis of the effect of a single parameter on the FSEed process and on the microstructural and mechanical characterization of the obtained specimens have been considered. Therefore, the purpose of this work is to develop a model based on simulative and mathematical techniques, able to predict the correct effect of the process parameters on the obtained piece, ensuring the extrusion of completely massive pieces. Indeed, the conditions for which the extrusion arises do not depend individually on the thermal history or on the deformations to which the scraps are subjected, but they are strongly linked by the relationship between these conditions.

The main original point of this approach is the development of a FEM model, implemented with a Fortran routine, to predict if, how and where the bonding phenomenon occurred. To build a robust technological window, different process parameters, namely the tool descent speed and the tool rotational speed, were considered. The final aim of this paper is to analyze the bonding phenomenon both from the thermal and the stress conditions generated during the FSE by the process parameters point of view. To do that, different available criteria for the analysis of the bonding quality were considered, to find the most appropriate for this new kind of extrusion, based on the bonding of the aluminum scraps. The first considered method was the simplest one, introduced by R. Akeret in 1972, which studied the extrusion quality only as a function of the maximum pressure in the bonding/extrusion chamber [20]. If this pressure exceeds a critical limit, experimentally determined, the welding can be assessed as completed. This relationship can be translated as follow:

(1) $$P=max\left(p_{i}\right)$$

Even if the simplicity of this approach, to the authors’ best knowledge, it has never been experimentally validated. Another criterion was introduced by Bourqui et al., which states that a good bonding is achieved if the ratio between the maximum pressure in the extrusion chamber (*P_w_*) and the pressure at the upper section of the matrix hole (*P_s_*) exceeds the value of 0.5 [21]:

(2) $$\frac{P_{w}}{P_{s}}>0.5$$

However, this criterion could not be applied with low extrusion ratios. Nowadays, one of the most used criteria for aluminum extrusion is the Donati–Tomesani model, reported in Equation (3) [22].

(3) $$K=\int_{A}^{}\frac{P}{\sigma }dA\geq Cost$$

In this equation, *K* identified a bonding index, linked to the integral extended to the welding area (*A*) of the ratio between the local hydrostatic pressure, *P*, and the Von Mises effective stress, *σ*. *Cost* is a critical value of the index, and it is able to guarantee a solid joint. The presence of the integral expressed considering the welding area means that the aim of this approach is to provide a single *K* as quality index for the overall joint, to be extended for the whole profile length. When the most significant parameter to be considered during the experimental or simulative campaign is not the length of the extrusion, but the time for which the contact between the material to be extruded takes place, an alternative criterion, proposed by Plata and Piwnik has to be applied [23]. It is based on the ratio between the local pressure (*p*) and the effective stress acting in the material (*σ_eff_*), along a generic path for a welding element, integrated on time. The value obtained must exceed a critical limit, to be experimentally determined. According to this criterion, the material bonding occurs if the parameter *w* reaches a limit value, called *w_lim_*:

(4) $$w=\int_{t}^{}\frac{p}{\sigma_{eff}}\cdot dt$$

In the present paper, the Piwnik and Plata bonding criterion was chosen, and a porous material was considered to simulate the starting workpiece.

## 2. Materials and Methods

A 3D FEM Lagrangian model was settled using the commercial software DEFORM V13, in which the scraps container, extrusion chamber, and rotating tool were treated as rigid objects made of AISI-1043 steel. The extruded metal scraps were represented as a single porous workpiece made of Aluminum-6061 and placed within the extrusion chamber. To condense the time required for the computation of the simulations, the initial tool descent and the compacting phase of the scraps were eliminated, but the initial density of the scraps was experimentally evaluated. Through experimental scraps pre-compaction tests, a maximum density of 2.11 g/cm^3^, equal to 78% of the base aluminum, was obtained by considering homogeneous scraps made by AA6061, which chemical composition is reported in Table 1.

**Figure 1:**
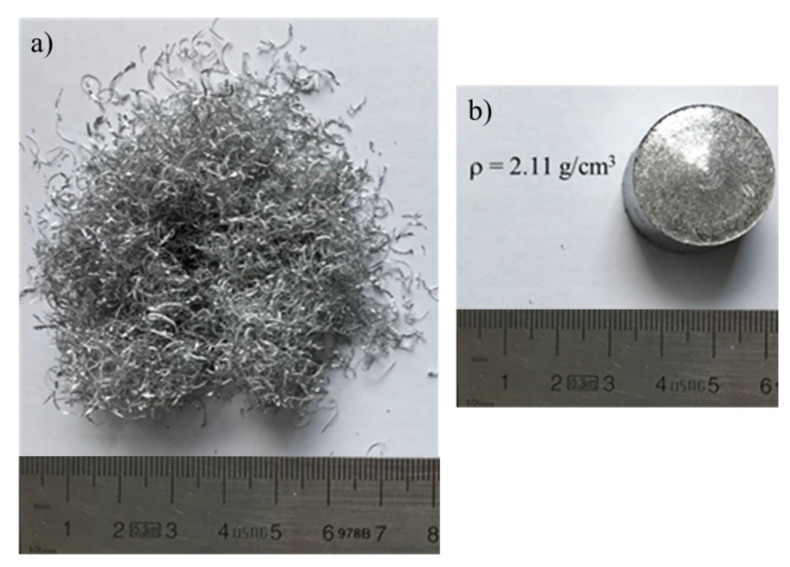
(**a**) Metal scraps dimension and (**b**) pre-compressed samples density.

The starting scraps and the obtained pre-compressed specimen are shown in Figure 1. This value was fixed for the workpiece initial condition for all the simulations.

**Figure 2:**
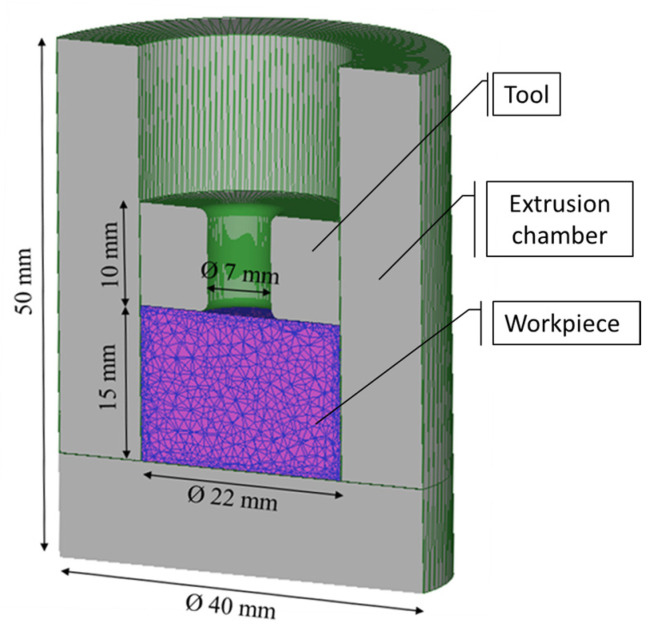
Mesh of the workpiece and geometry of all simulated structure.

Data for flow stress and thermal properties of both the steel instrumentation and aluminum scraps were selected from the software library database, and the porous workpiece was meshed using 50,000 tetrahedral elements, as shown in Figure 2.

**Figure 3:**
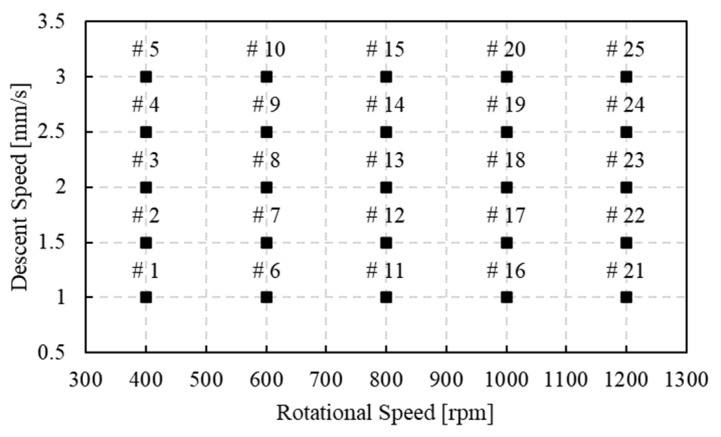
Tool descent speed (S) and tool rotational speed (F) combinations considered for the simulations. The symbol # followed by the numbers indicates the number of the combination formed considering the rotational speed and the descent speed indicated by the axis.

The constitutive model chosen for both the materials was the flow stress function reported in Equation (5):

(5) $$\sigma =\sigma (\varepsilon ,\dot{\varepsilon },T)$$

in which σ is the flow stress, ε is the strain, $\dot{\varepsilon }$ is the strain rate, and *T* indicates the temperature. The relationship between these parameters was given by the interpolation automatically performed by the software, basing on the experimentally calculated values. It is important to underline that, in Deform, the porous objects are treated the same as plastic objects, except for the material density, which is calculated and updated for every node at every step of the simulation. For this reason, the characteristics of the porous materials, such as the limiting strain rate and the flow stress, must be referred at the fully dense state. Moreover, in the developed model, the heat was chosen to be generated only by the friction generated between the porous material and the tool. The simulations were conducted varying the main FSE process parameters, i.e., the tool descent speed (S) and the tool rotational speed (F) as reported in Figure 3. All the combinations between these S and F were considered by conducting 25 different simulations.

The thermal parameters of the FEM model were held constant, and the values listed in Table 2 were utilized. These values derived from a combination between the sensitivity analysis proposed for the Friction Stir Welding process [24] and the same analysis conducted for the Friction Stir Extrusion process [25]. The simulations were stopped when a die displacement equal to 3.5 mm was reached, since this stroke demonstrated to correctly represent the steady-state condition.

To define if S or F influenced the thermal input on the workpiece at the steady-state conditions, the ANOVA technique was applied with an α equal to 95%. Table 3 presents the *p*-values pertaining to the impacts of S and F on the maximum temperature achieved during the FSE process. It is important to underline that, in statistical hypothesis testing, *p*-values are used to determine the statistical significance of an observed effect. A low *p*-value (below a predetermined threshold, in this case equal to 0.05) indicates a statistically significant relationship or effect. Conversely, a high *p*-value implies that the observed effect may be attributable to random chance rather than a meaningful relationship.

**Figure 4:**
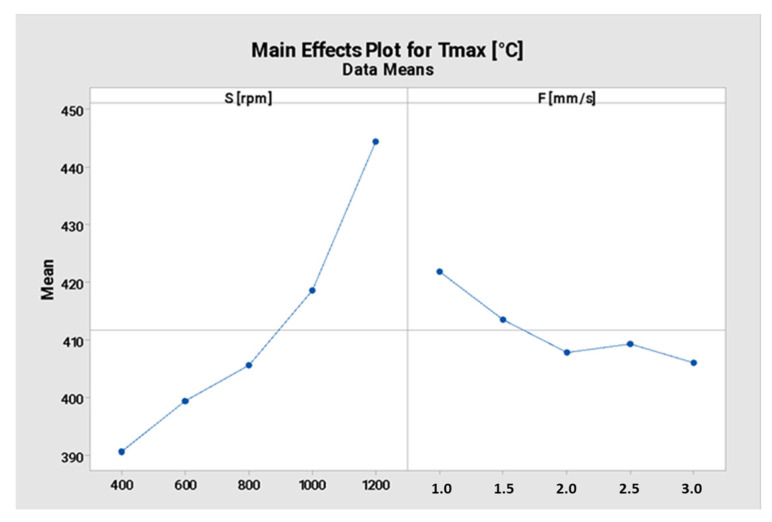
Main effects plot for the maximum temperature reached just below the tool.

In this case, the analysis revealed that the *p*-values associated with the factors tend towards 0.000. This indicates a statistically significant relationship between the tool rotational speed and the maximum temperature reached just below the tool. Figure 4 shows the main effects plot depicting the relationship between the factors and the response variable, specifically the maximum temperature just below the tool. The plot clearly displays whether there are any relationships, either existing or not existing, between the factors and the responses.

Based on this finding, it can be concluded that as the tool rotational speed increases, there is an increased likelihood of achieving maximum temperatures equal to or greater than 400 °C, regardless of the tool descent speed. This is beneficial for the FSE, as it ensures the attainment of suitable temperature parameters.

Despite of the strong relationship between the temperature distributions and the tool rotational speed, in the authors opinion, the maximum temperature reached just below the tool cannot be considered as the unique parameter of the performance quality of the FSE process. In fact, ANOVA has proved to be very effective in finding a relationship between the process parameters and the thermal history of the extruded, but it does not prove to be useful in predicting the bonding conditions that may occur inside the FSEed piece. Furthermore, it is known that the bonding phenomenon depends on the distribution of stresses in the material and on the time for which these stresses act. For these reasons, it is necessary to consider a method which can involve the joint consideration of the reached temperature and of the stress distribution within the porous workpiece. Consequently, for analyzing if and how the solid-state welding of the metal scraps occurred, the Piwnik and Plata criterion was considered. The occurrence of material bonding is determined by the criterion that the parameter *w* reaches a value, referred to as $w_{lim}$, which is dependent on the temperature and on the considered material. The parameter w is defined as the ratio between the local pressure (p) and the effective stress acting in the material ($\sigma_{eff}$), integrated along the time for which the contact takes place (t), as:

(6) $$w=\int_{0}^{t}\frac{p}{\sigma_{eff}}\cdot dt$$

Considering the total number of the simulation steps, the expression reported in the Equation (6) was converted in a sum:

(7) $$w_{i,n}=\sum_{j=1}^{n}{\left(\frac{p}{\sigma_{eff}}\right)}_{i,j}\cdot \Delta t_{j}$$

where:

- *n* is the current step number;
- *j* is the generic *j*-th simulation step;
- *i* is the generic *i*-th element;
- Δ*tj* is the duration of the *j*-th simulation step.

To apply this criterion to the FSE process, the local pressure appearing in the numerators of Equations (6) and (7) were replaced by the *σ_mean_* acting in the porous material. This choice was made because the Piwnik and Plata criterion is used to study the bonding conditions in a traditional extrusion process, considering the pressure generated between the parts of material that come into contact, for example after passing through the matrix holes. On the contrary, in the case of the FSE, the starting material is composed by scraps, even if in the simulation was considered as a single porous material, so it was impossible to calculate the local pressure deriving from the interaction between the individual chips.

In the literature, a procedure for the $w_{lim}$ calculation as a function of the temperature is already present, based on a coupled experimental-simulative strategy applied to a traditional rolling process [26,27]. E. Ceretti et al. built an experimental interpolation curve to define $w_{lim}$ as a function only of the steady-state temperature (*T*) defined as [26]:

(8) $$w_{lim,i}=4.9063e^{-0.0017\cdot T_{i}}$$

where *i* is the generic *i*-th element of the mesh. This equation was verified in E. Ceretti et al. only for *T* > 320 °C [26], but this temperature interval totally agrees with the entire temperature window considered by the authors in the FSE process, since the minimum peak temperature reached at steady-state condition was equal to 386 °C.

**Figure 5:**
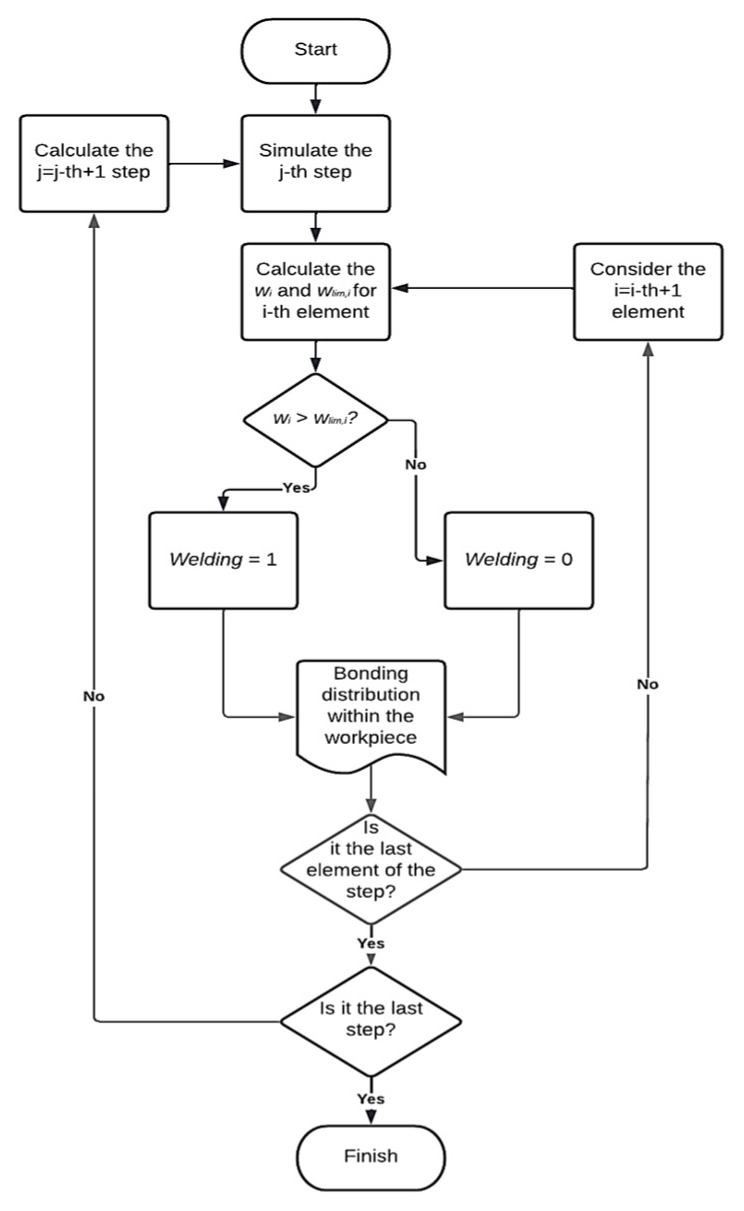
Flowchart of the Piwnik and Plata application embedded in the simulative process.

To better identify where the bonding took place, a welding parameter was introduced. This parameter, initially set equal to 0, became equal to 1 when w reached the $w_{lim}$ value. After introducing the Piwnik and Plata criterion, the FEM model was updated, and a new set of simulations were conducted. In these simulations, the *w*, $w_{lim}$, and the welding parameter values were calculated for each element at each step of the simulation, up to reach the stop criterion (Figure 5). Indeed, it is necessary to calculate these parameters for each single element at each step because they are linked to the distribution of temperature and stresses, which are not uniform within the material to be extruded. For this reason, for each step it is possible to monitor the bonding situation of the entire porous material considered during the whole simulation. To do that, a suitable Fortran routine was developed for studying how *w* and $w_{lim}$ vary as the temperature and the working conditions vary. The bonding parameters were stored in user-defined variables associated to each element.

## 3. Results

**Figure 6:**
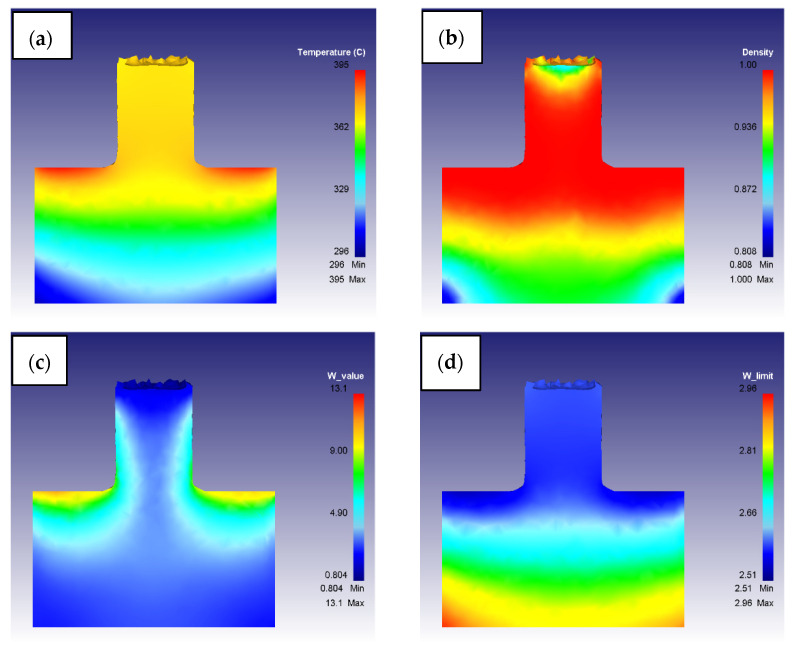
Graphical results of a FEM model simulation (S = 400 rpm and F = 1 mm/s): (**a**) Temperature, (**b**) Density, (**c**) *w*, and (**d**) *w_lim_*.

After having performed all the simulations, temperature, density, and both *w* and *w_lim_* maps of the workpiece were extracted for each combination of parameters. As an example, the results obtained considering the first process parameter couple (S = 400 rpm and F = 1 mm/s) are reported in Figure 6.

**Figure 7:**
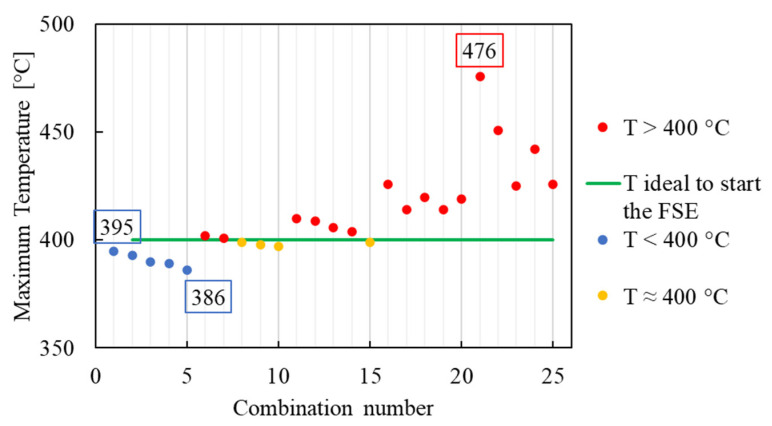
Maximum temperature reached below the tool at the steady-state condition for all the simulated combination of parameters. The number of each combination is the same as shown in Figure 3.

The maximum temperatures reached during the simulations just below the rotating tool are reported in Figure 7.

**Figure 8:**
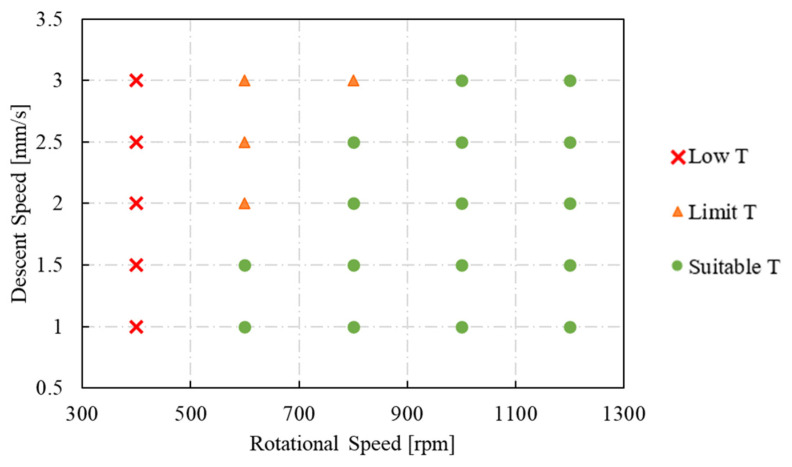
Diagram as a function of the rotational speed (S) and the descent speed (F) of the tool analyzing if the conditions guarantee temperatures below the tool less than, equal to, or greater than 400 °C.

As already mentioned in the introduction section, the aluminum alloys are characterized by a low softening temperature, which is around 400 °C. For the FSE process, it is important to never reach the scraps melting temperature, since FSE is a solid-state process. This leads to consider a temperature between 400 °C and 660 °C as a suitable window for the FSE process of the aluminum. From the temperature distribution reported in Figure 7, it is clear that almost all the considered combinations of parameters satisfy this requirement. The maximum temperature (476 °C) was reached for S equal to 1200 rpm and F equal to 1 mm/s (combination number 21), whilst the minimum peak temperature (386 °C) was registered for the combination number 5 (S = 400 rpm and F = 3 mm/s). Therefore, considering only the thermal input of the process, all the combinations of parameters from the number 6 (S = 600 rpm and F = 1 mm/s) to the number 25 (S = 1200 rpm and F = 3 mm/s) would ensure the correct progress of the extrusion process. The schematic representation of the conditions obtained from the simulations, as a function of the combinations of parameters, considering only the maximum temperature reached below the tool during the simulations, is reported in Figure 8.

As already said, in the author’s opinion, the maximum temperature extrapolated from a simulation cannot be considered as the unique parameter of the performance quality of the FSE process since it is well known that the bonding phenomenon depends on the distribution of stresses in the material and on the time for which these stresses act, as already explained in the previous paragraph. Thus, in order to consider the simulation process as a suitable tool to predict if the selected process parameters led to a completely massive extruded piece, it was necessary to make the simulation ever closer to what happens in reality. To do that, it was important to consider not only the thermal evolution of the system, but also the stress conditions occurring within it. In this research, the stress conditions were evaluated calculating and comparing *w* and *w_lim_* values for each element of the mesh at the end of each simulation step.

**Figure 9:**
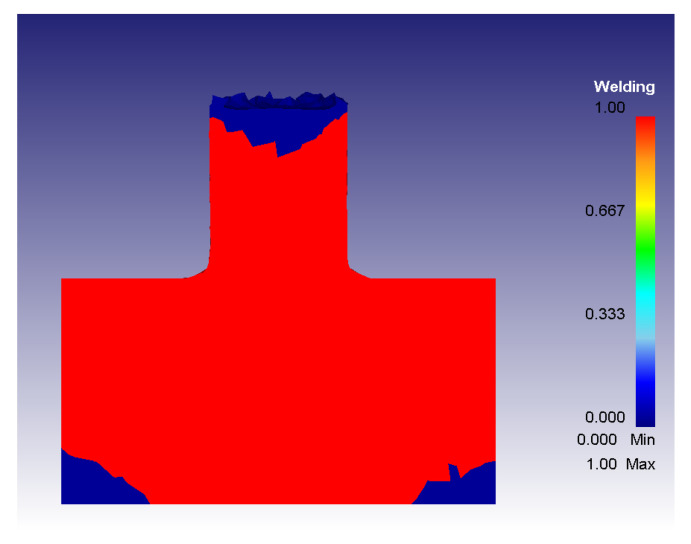
Welding variable map obtained with S = 400 rpm and F = 1 mm/s.

When the *w* value becomes greater than the *w_lim_* value, it means that bonding is taking place according to the Piwnik and Plata criterion. For this reason, in the developed Fortran routine, the bonding condition occurrence was identified defining a new variable (namely *Welding*) whose value pass from 0 to 1 in case of bonding condition occurrence. In this case, the element for which this condition was verified is colored in red as shown in Figure 9. The blue color indicates the part of the material in which the stress conditions and the temperature distribution cannot ensure the bonding of the scraps. By means of this solution, it is possible to identify when and where the material bonding really takes place.

**Figure 10:**
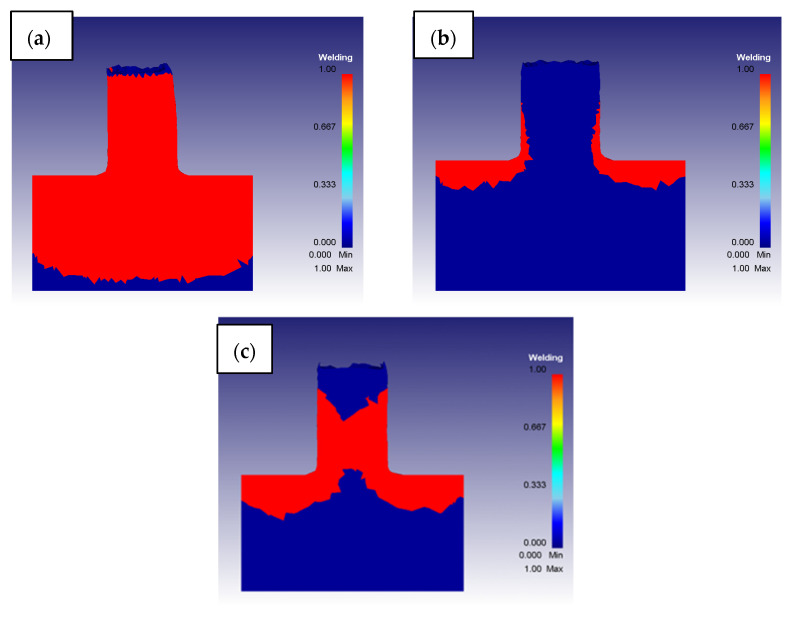
Different bonding conditions: (**a**) Completely bonded with S = 1200 rpm and F = 1 mm/s, (**b**) Completely not bonded with S = 600 rpm and F = 2.5 mm/s, and (**c**) Limit condition with S = 800 rpm and F = 2 mm/s.

By analyzing the results obtained at the end of all the simulations, it was possible to identify three different global conditions: bonding, not bonding, and limit condition, as reported in Figure 10 for the different working parameters combinations. The situation of complete bonding of the metal scraps (Figure 10a) can be distinguished from the situation in which the bonding of the material was never reached before its extrusion (Figure 10b).

The limit condition foresaw the coexistence of already completely bonded material and still porous metal scraps (Figure 10c). This condition would give the possibility of obtaining extruded pieces whose massive condition affects only the external surface, but with still internal parts of material not bonded. This situation could lead to many problems if not identified in time, because the extruded piece could be characterized by a much more fragile behavior than a completely massive FSEed sample.

To overcome these problems, it is fundamental to precisely define the boundary condition zone, to choose a combination of parameters that surely falls within this area.

**Figure 11:**
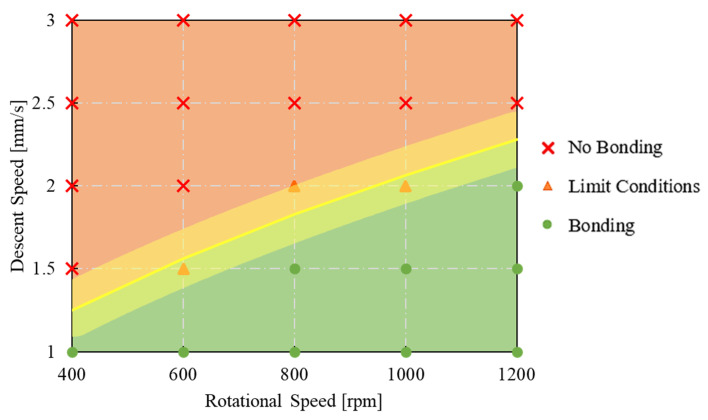
Bonding diagram as a function of tool descent speed and rotational speed in view of the Piwnik and Plata criterion.

This can be conducted by looking at the diagram reported in Figure 11 showing the experimental results in terms of bonded or not bonded specimens. The red area represents the no bonding zone, the green area indicates the bonding zone, and the yellow area represents the limit condition zone.

**Figure 12:**
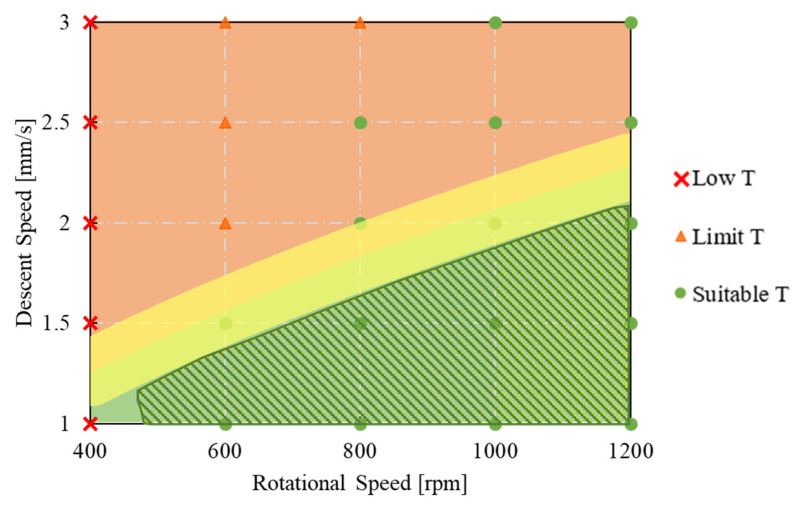
Bonding diagram as a function of tool descent speed and rotational speed in view of the Piwnik and Plata criterion and the temperature simultaneously (technological window).

The results show how it is correct to expect that the combinations of the process parameters play an important role in defining the stress conditions at which the metal scraps can be bonded, and the slower the rotational speed, the slower the descent speed must also be. By merging the graphs reported in Figure 8 and the bonding areas defined in Figure 11, it was possible to identify the combinations of parameters that ensured the fulfilment of both requirements necessary for the correct execution of the FSE process. In the dashed green area in Figure 12, both the thermal limits, i.e., parameters that guarantee temperatures below the tool equal to or greater than 400 °C, and stress conditions compatible with the complete bonding of the scraps were respected.

Therefore, it can be stated that by moving within this area, the FSE process can run correctly, and the pieces obtained will be completely massive.

Analyzing the results, it was evident that considering either the thermal history linked to the process, or the stress conditions of the workpiece individually would lead to an incorrect forecast of the process performances. For this reason, the combined methodology developed can be considered a valid tool for identifying the combination of process parameters (technological window) that can be used for the FSE of any aluminum alloy scraps, considering however that the *w_lim_* relationship must be redefined case by case.

## 4. Conclusions

In the present research, the bonding occurrence in the FSE process was considered and a combined simulative and mathematical model was developed. Firstly, a robust FEM model was built considering aluminum scraps worked with different tool rotational and descent speeds. Secondly, the Piwnik and Plata bonding criterion was applied to analyze the bonding phenomena as a function of the stress conditions and temperature distribution resulting in the workpiece at its steady state.

In conclusion, this paper demonstrates that:

- The FEM model developed in this study can effectively predict the temperature distribution experienced by scraps during the entire FSE process.
- The Piwnik and Plata criterion is well suited for simulating the effectiveness of this technology.
- The Fortran routine developed in this study can automatically calculate the Piwnik and Plata terms for each element.
- The combination of the maximum temperature achieved below the tool and the Piwnik and Plata results can serve as a reliable approach to accurately forecast the occurrence of material bonding.

The combined method proposed in this study enabled the establishment of a technological window for achieving a dependable FSE process, by determining the appropriate combination of tool rotational and descent speeds. The results obtained demonstrate that the conditions necessary for achieving solid bonding are contingent upon both the temperature and stress conditions experienced by the scraps during the FSE process.

## Figures and Tables

**Table 1:** Chemical composition of the AA6061.

Al	Mg	Si	Fe	Cu	Cr	Mn	Zn	Ti
Bal.	0.9	0.61	0.33	0.25	0.17	0.05	0.02	0.02

**Table 2:** Parameters used for the simulations.

Parameters	Value
Friction coefficient aluminum-tool	0.60
Thermal conductivity	450.00 N/(s∙°C)
Aluminum emissivity	0.25
Steel emissivity	0.70
Heat transfer coefficient aluminum-tool	11.00 N/s/mm/°C
Heat exchange with the environment	0.02 N/s/mm/°C
Mechanical conversion to heat	0.80

**Table 3:** ANOVA results for the maximum temperature reached below the tool during the simulations.

**Factor Information**					
**Factor**		**Levels**	**Values**		
F [mm/s]		5	1.0; 1.5; 2.0; 2.5; 3.0		
S [rpm]		5	400; 600; 800; 1000; 1200		
**Analysis of Variance**					
**Source**	**Degree of Freedom**	**Adjusted Sum of Squares**	**Adjusted Mean Squares**	**F-Values**	***p*-Values**
F [mm/s]	4	793.0	198.26	2.76	0.064
S [rpm]	4	8753.0	2188.26	30.42	0.000
Error	16	1151.0	71.94		
Total	24	10,697.0			

## Data Availability

Data are to be considered confidential and will be provided after a suitable request.
